# Monitoring Building Deformation with InSAR: Experiments and Validation

**DOI:** 10.3390/s16122182

**Published:** 2016-12-20

**Authors:** Kui Yang, Li Yan, Guoman Huang, Chu Chen, Zhengpeng Wu

**Affiliations:** 1 School of Geodesy and Geomatics, Wuhan University, Wuhan 430079, China; yangkui726@126.com; 2 Tianjin Institute of Surveying and Mapping, Tianjin 300381, China; chenchu622@163.com (C.C.); zhengpeng_wu@163.com (Z.W.); 3 Chinese Academy of Surveying and Mapping, Beijing 100830, China; Huang.guoman@casm.ac.cn; 4 Tianjin Remote Sensing Center, Tianjin 300381, China

**Keywords:** building deformation monitoring, InSAR, accuracy index, validation test, OLS regression analysis, measurement of error analysis

## Abstract

Synthetic Aperture Radar Interferometry (InSAR) techniques are increasingly applied for monitoring land subsidence. The advantages of InSAR include high accuracy and the ability to cover large areas; nevertheless, research validating the use of InSAR on building deformation is limited. In this paper, we test the monitoring capability of the InSAR in experiments using two landmark buildings; the Bohai Building and the China Theater, located in Tianjin, China. They were selected as real examples to compare InSAR and leveling approaches for building deformation. Ten TerraSAR-X images spanning half a year were used in Permanent Scatterer InSAR processing. These extracted InSAR results were processed considering the diversity in both direction and spatial distribution, and were compared with true leveling values in both Ordinary Least Squares (OLS) regression and measurement of error analyses. The detailed experimental results for the Bohai Building and the China Theater showed a high correlation between InSAR results and the leveling values. At the same time, the two Root Mean Square Error (RMSE) indexes had values of approximately 1 mm. These analyses show that a millimeter level of accuracy can be achieved by means of InSAR technique when measuring building deformation. We discuss the differences in accuracy between OLS regression and measurement of error analyses, and compare the accuracy index of leveling in order to propose InSAR accuracy levels appropriate for monitoring buildings deformation. After assessing the advantages and limitations of InSAR techniques in monitoring buildings, further applications are evaluated.

## 1. Introduction

Building deformation has become one of the most important threats to property and lives in many cities worldwide. For example, there are many active landslides in Europe that cause deformation of buildings, resulting in severe damage [1,2,3,4,5]. In China, along with rapid economic development, construction of underground projects such as subways and foundation pits are increasing and are the main factors behind building deformation [6]. Therefore, monitoring the stability of buildings is becoming fundamental for prevention of accidents.

Until now, leveling measurement was the conventional method for monitoring building deformation. By establishing leveling points on buildings, and through precise, repeated observations, this technique can directly measure building motion directly at high accuracy [7]. Table 1 presents leveling technique monitoring levels, the corresponding accuracy and applicability. The first level with a precise accuracy of 0.2 mm is used for the monitoring important ancient buildings; the second level at an accuracy of 0.7 mm is applied in monitoring buildings with ground foundation design of A and B, and it is sufficient in most applications; and the third level at the accuracy of 2.1 mm can be used for monitoring buildings with ground foundation design of B and C [7]. However, this method faces many challenges, including the cost and feasibility in regard to time and space given the increasing demand for building deformation monitoring data. Synthetic Aperture Radar Interferometry (InSAR) is proposed and applied to overcome these challenges.

Because InSAR can monitor ground deformation caused by earthquakes with millimeter accuracy [8], it has received enormous attention from scholars and researchers in the field of geosciences. Advanced multi-temporal interferometric techniques in particular offer high accuracy deformation results using time series analysis of SAR datasets, Persistent Scatterer Interferometry (PSI) [9,10], Small Baseline Subset Approach (SBAS) [11], Coherent Pixels Technique(CPT) [12] and Interferometric Point Target Analysis (IPTA) [13], have been effectively applied in many fields, such as glacier movement [14], volcanic activity [15], urban subsidence movement [16], landslides instability [2], and underground mining activity [17]. Many experiments have been conducted to validate displacement accuracy within the millimeter-to-sub millimeter levels. For example, Wegmüller et al. compared the IPTA result achieved from TerraSAR-X with leveling data. An overall good correspondence confirmed the utility of the TerraSAR-X data and the applied IPTA methodology [18]. Luo et al carried out Tianjin ground monitoring experiments. using 172 leveling points and some GPS data to validate the InSAR results from X-band and L-band, demonstrated the millimeter accuracy of the PSI technique [19]. The experiment carried out by Qin and Perissin, using four corner reflectors installed on a construction site of Hong Kong, demonstrated the millimeter accuracy of the PSI technique in real application scenarios with when compared in-situ leveling records [20]. InSAR applications have also been extended to monitor buildings [1,3,4,5,21,22]. Ciampalini et al. used five different sensors (including ERS-1/2, ENVISAT, RADARSAT-1, COSMO-SkyMed and TerraSAR-X) and PSI technique to successfully evaluate the building deformation velocities in the San Fratello municipality, Italy, which was affected by two landslides occurring in 1922 and 2010. There was a very good agreement between field survey damage assessment and the building deformation velocity map obtained through COSMO-SkyMed. These results also confirmed that the most intensely damaged buildings during the landside of 2010 were characterized by residual movements [1]. Frattini et al. used both the displacement data from PSI and the damage degree from field surveys to conclude that the degree of damage increases with increasing displacement rate in general [3]. Bianchini et al. in [4] proposed a methodology based on PSI results in the X-band to realize a single building-scale analysis of differential settlement, thus overcoming the limitations related to the point-wise nature of PS information, with good accordance between estimated building deformations and damage evidence from field surveys.

However, previous research focused on InSAR accuracy has mainly concerned InSAR applications over large areas; thus, the conclusions cannot be used directly for monitoring single building given the law of error propagation [23], which implies that the accuracy of ground subsidence for large areas is larger than the monitoring accuracy of buildings due to relative distance factors. In addition, previous research on application of InSAR data on building deformation was analyzed from a qualitative viewpoint using only field survey results; no quantitative analyses have been performed comparing the InSAR accuracy for monitoring buildings with in-situ leveling records.

To fill this gap in the literature and extend this research, we conducted a building monitoring experiment in the urban area of Tianjin, China, applying InSAR technique and comparing the obtained measure values with tradition leveling data to quantitatively demonstrate the millimeter accuracy of InSAR in practical building applications. With the aim of raising the confidence and accuracy of the InSAR techniques for building monitoring, two landmark buildings, the Bohai Building and the China Theater, both located near a foundation pit, were concurrently monitored with optical leveling and InSAR. After solving for the diversity between these two methods through spatial interpolation methods, the analytical results from InSAR were compared to in-situ leveling data by means of regression analysis and statistical analysis of the measurement error. Finally, the advantages and limitations of the InSAR technique applied to this building monitoring project were fully evaluated, in order to provide some useful references for similar applications in the future.

## 2. Experimental Area and Materials

### 2.1. Experimental Area

**Figure 1:**
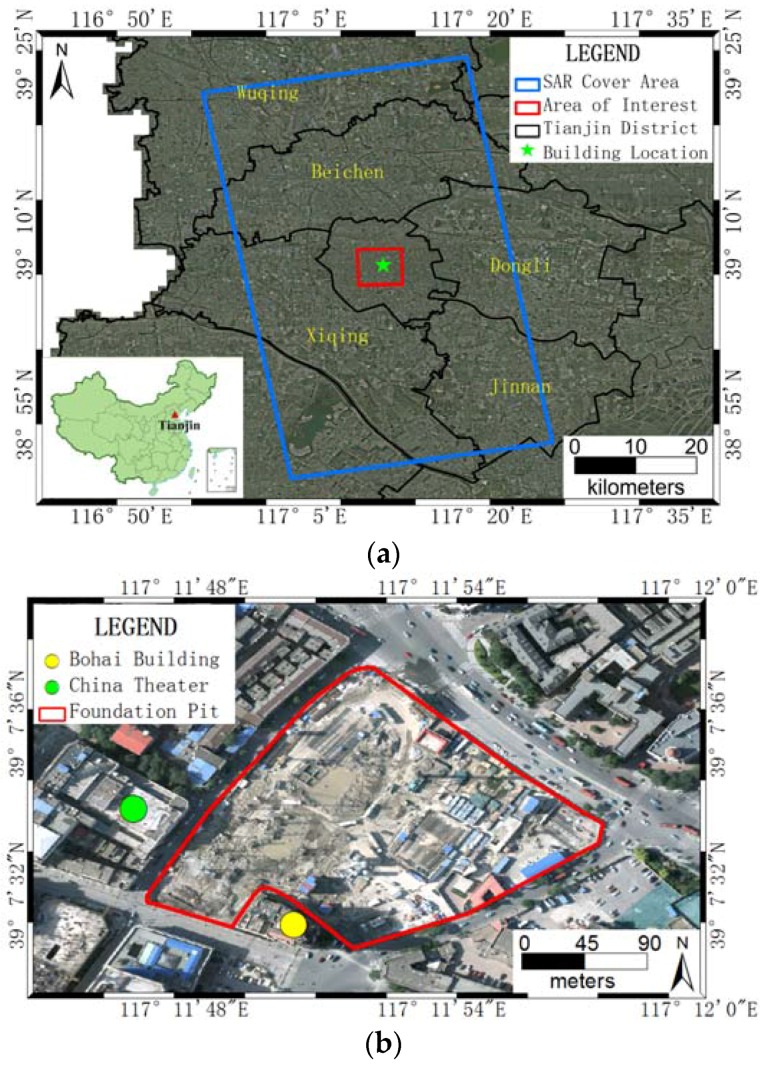
(**a**) Location of the AOIand coverage of SAR data for subsidence monitoring in the Tianjin region, with aerial ortho-photo of 1 m as background; and (**b**) location of two experimental buildings near a foundation pit, with aerial ortho-photo of 0.2 m as background.

The experimental area of interest is located in the city center of Tianjin, China. In Figure 1a, the covering area of Synthetic Aperture Radar (SAR) is indicated by a blue rectangle, and the area of interest (AOI) is marked with a red rectangle. Two buildings located near a foundation pit (the green star in Figure 1a) were selected for monitoring with InSAR; in-situ leveling data were used to validate the monitoring results. The Bohai Building shown in Figure 1b, a historical architectural landmark building in Tianjin, was selected; the other landmark structure selected was the China Theater, a large-scale entertainment venue for the performing arts.

### 2.2. IPTA Analysis for Buildings

Available satellite radar dataset over the AOI in Tianjin consists of ten SAR images acquired in the X-band by the TerraSAR-X satellite of DLR (Germany Space Agency), for a time interval from May 2014 to December 2014. The acquisition satellite parameters of the available SAR images are listed in Table 2. 

The TerraSAR-X dataset was processed using the IPTA approach, which extends the PSI approach. It overcomes the limitations imposed by spatiotemporal decorrelation and atmospheric disturbances through the analysis of only selected stable points [18]. The main steps of IPTA are as follows: refinement co-registration with exterior digital elevation model (DEM), interferogram stack based on the multi-image principle, Permanent Scatterer (PS) selection using low diversity spectral and temporal criteria, spatial and temporal phase unwrapping, atmospheric phase screen (APS) estimation with a spatial and temporal filtering strategy, deformation velocity estimation, and geocoding with imaging models and reference points [24,25].

In order to obtain serious building deformation with small dataset in our case, some improvements are used in the IPTA analysis. One important improvement is in the step of interferogram formation: a multi-image principle was applied in order to obtain a stack with 30 interferograms from ten SAR images [26]. The second vital improvement is that a joint strategy was developed for the selection of PS, a sub-correlation method with a threshold of 0.35 and amplitude deviation measure with threshold value of 1.4 were used to select PS with high-spatial or high-temporal coherence, then a spatial average method of the interferograms was used to filter inaccurate points, in order to improve the quality of PS [24,26,27]. The third improvement is that a simple linear deformation model is used in the step of spatial-temporal phase unwrapping to get linear deformation in the AOI.

**Figure 2:**
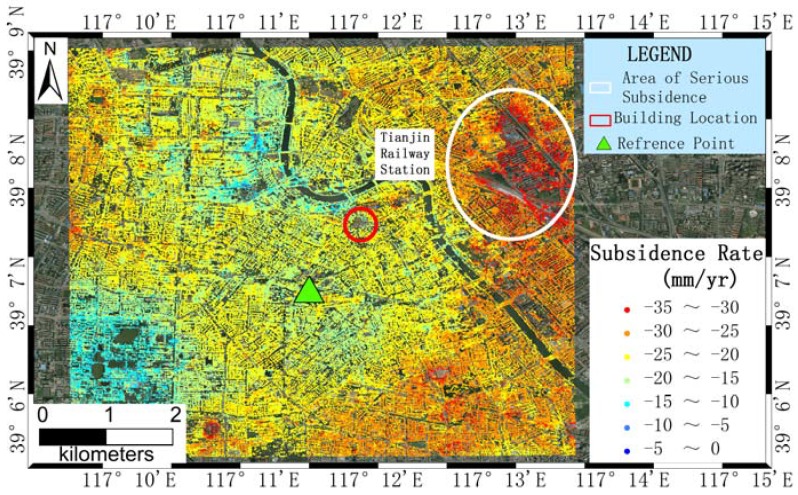
Linear Subsidence velocity with ranges from −35 to 0 mm/year from Interferometric Point Target Analysis (IPTA)process, the area near Tianjin Railway Station have subsidence more than 30 mm/year, and the location of two selected buildings have subsidence value of approximately −20 mm/year. The background is aerial ortho-photo of 1 m.

The final annual velocity of displacement in the AOI after the correction of the overall offset caused by reference point (marked with green triangle) is displayed in Figure 2. The overall annual velocity around our AOI varied from −35 to 0 mm/year, and most of the analyzed areas displayed velocities of approximately −20 mm/year. Some partial regions have velocities smaller than −30 mm/year, for example the area near the Tianjin Railway Station (marked with a white ellipsis). The area corresponding to the location of two buildings (marked with a red ellipsis) have subsidence velocities of approximately −20 mm/year.

**Figure 3:**
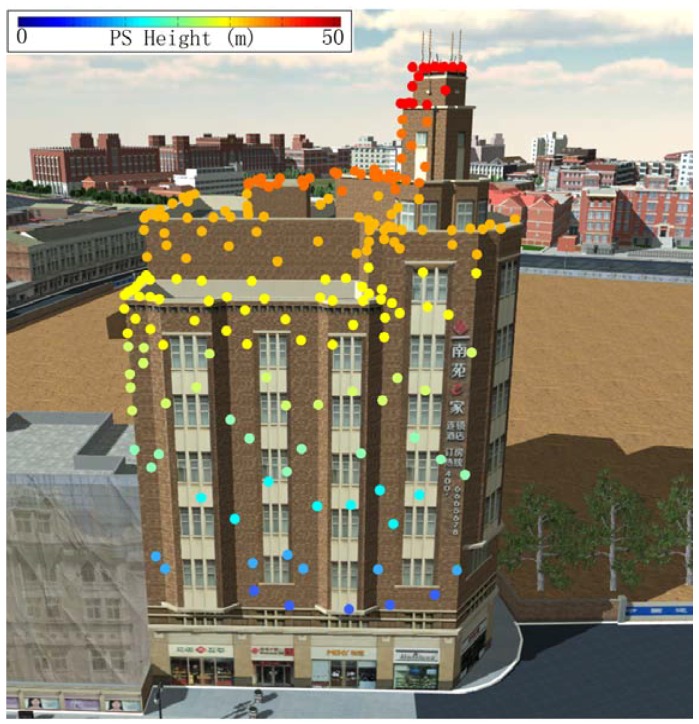
Three-dimensional representations of extracted Permanent Scatterer (PS) point geocoded on precise 3D models for the Bohai Building. All the PS satisfying the stability criteria during IPTA processing (sub-correlation coefficient larger than 0.35 and amplitude deviation measure larger than 1.4) have been located on roof and facades toward the satellite, in the monitoring period from May to December 2014.

**Figure 4:**
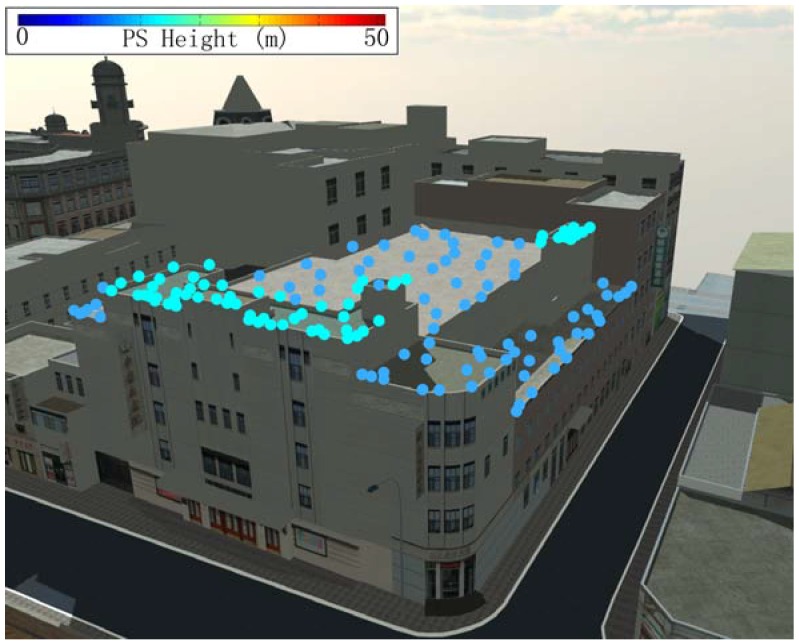
Three-dimensional representations of extracted PS point geocoded on precise models for the China Theater. All the PS satisfying the stability criteria during IPTA processing (sub-correlation coefficient larger than 0.35 and amplitude deviation measure larger than 1.4) have been located on roof, in the monitoring period from May to December 2014.

A strategy for extracting building PS was applied for further building deformation analysis in consideration of complex urban environment. A rich and precise geographic database containing urban buildings mapped to a scale of 1:2000 was used to recognize the building to which each PS corresponds. Overlay analysis was used to match geocoded PS and the geographic database to obtain primary building PS [27]. Inaccurate buildings PS were further filtered by considering the features of buildings in SAR images. Figure 3 and Figure 4 show the three-dimensional (3D) representations of the locations of extracted PS point geocoded on precise 3D models for the Bohai Building and the China Theater, during the monitoring period from May to December 2014. The stabilities of all extracted PS were in the range of selecting PS during IPTA process. Figure 3 shows 117 PS located on the roof, and on both facades of the Bohai Building. In Figure 4, 68 PS are located on the roof of China Theater. These PS in the two building are used for the leveling validation test.

### 2.3. Leveling Validation Test

The leveling deployment procedure consisted of two steps: (1) fix the proper locations for the buildings; and (2) high-frequency monitoring. 

Selection of the best site was carried out taking into account two basic requirements:

(1) The leveling points be installed and monitored by the validation team for the whole duration of the campaign (almost six months).

(2) The leveling points must be located on the crucial areas of the buildings, such as corners and facades, approximately ten meters apart [7].

**Figure 5:**
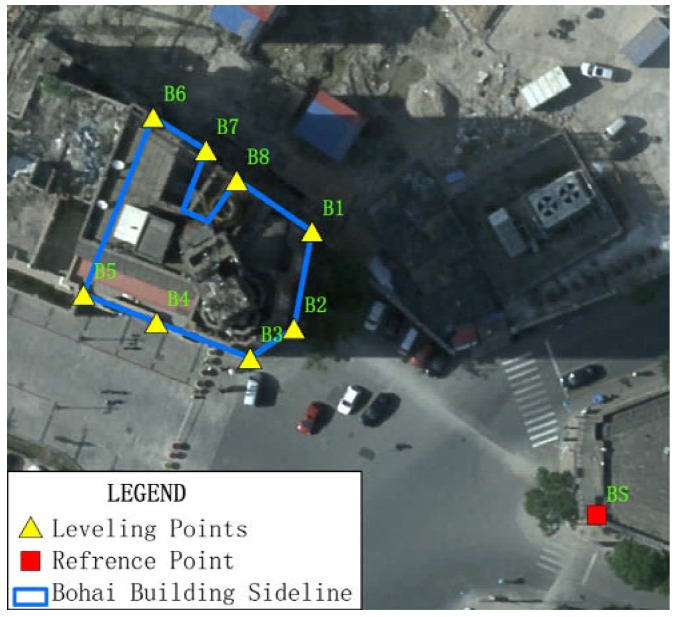
Locations of Leveling points and reference points for the displacement monitoring of the Bohai Building using leveling technique, with aerial ortho-photo of 0.2 m as background.

**Figure 6:**
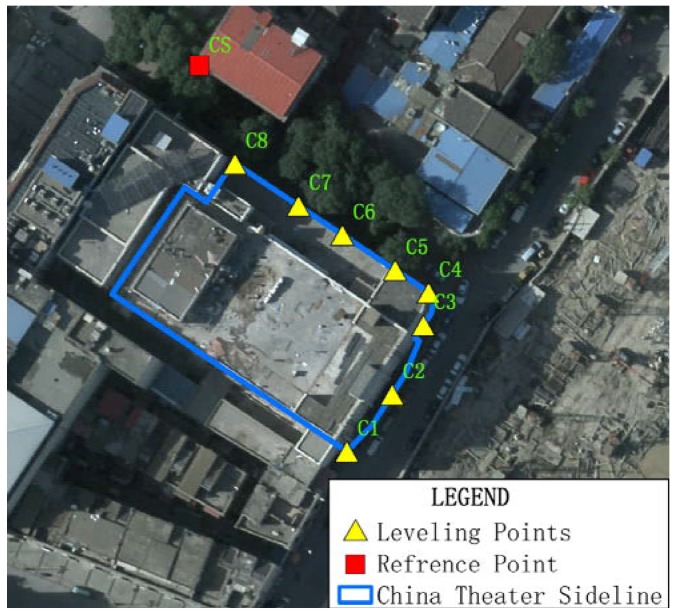
Locations of Leveling points and reference points for the displacement monitoring of the China Theater using leveling technique, with aerial ortho-photo of 0.2 m as background.

For the validation test, a set of leveling points was installed on the Bohai Building and the China Theater, while the points installed separately on nearby buildings served as reference points. During the construction of the foundation pit, their displacements were record by a survey team. We used these optical leveling results to validate displacement values extracted from InSAR data. In the case of the Bohai Building, eight points were installed for the test and were sequentially named B1 to B8. The reference point for the Bohai Building was named BS (Figure 5). In the case of the China Theater, eight points were also installed for validation, and they were named C1 to C8, and the reference point was named CS (Figure 6). 

For this study, a six-month validation period, from May to December 2014, was selected. High-accuracy observations with 0.7 mm were taken to monitor the displacement of these two buildings. The reference points (BS and CS) were used as starting points for leveling tasks. The cumulative displacements of these 16 points (B1–B8, C1–C8) are shown in Table 3.

## 3. Methodology

We applied an operative procedure to resolve the disparity between subsidence measurements extracted from InSAR and in-situ leveling, and two analysis methods are proposed for our comprehensive validation analysis. 

### 3.1. Displacement Direction Diversity Process

**Figure 7:**
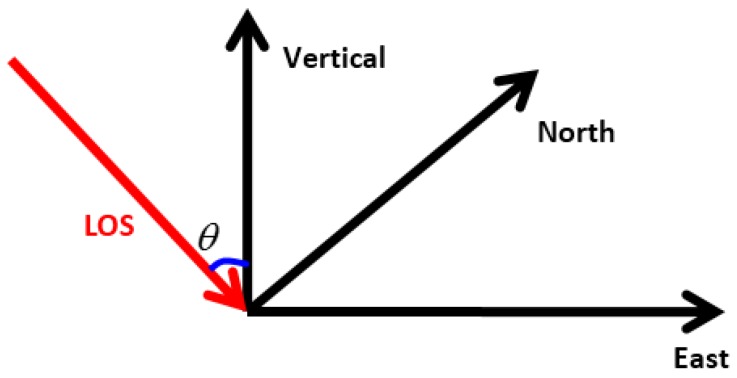
Monitoring Result Direction Difference between InSAR (LOS direction) and leveling task (vertical direction) for the reason of SAR side-look geometry.

Because of the right-side looking geometry, the InSAR estimate subsidence is sensitive only towards its light of sight (LOS) direction. However, the leveling task performed in the ground survey was in the vertical direction. The difference in direction between InSAR and leveling is shown in Figure 7.

Since in the current experiment only one ascending stack was acquired, the movement along the vertical direction cannot be derived directly. Deformation in the Bohai Building and the China Theater was caused mainly by the nearby foundation pit, supporting our assumption that the subsidence in LOS is caused by vertical displacement. Thus, to validate the accuracy of InSAR, InSAR subsidence must be converted to the vertical direction with the help of an incidence angle θ to make it comparable to leveling measurements [20,28].

### 3.2. Spatial Diversity Process

**Figure 8:**
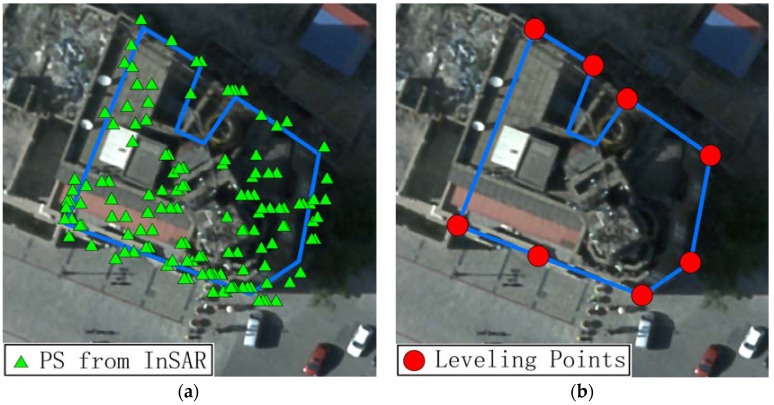
Spatial Diversity between InSAR and leveling in the Bohai Building with aerial ortho-photo of 0.2 m as background: (**a**) more than one hundred PS were located whole building from the vertical view; and (**b**) eight Leveling Points were located on edges of the Bohai Building.

For the reason of different formation mechanism between building PS and leveling points, there are major differences in the aspects of spatial distribution and point numbers. Referring to [29], the physical nature of PS in buildings are roofs, simple dihedrals and trihedrals, thus building PS are mostly located on the roof and both building facades toward the satellite, whereas the leveling points were fabricated according to the specifications for the given locations [7], and were located on corners or edges of building. The Bohai Building serves as a good example to analyze the spatial diversity here. The distribution of the monitoring PS from InSAR and leveling points are shown in Figure 8. The number of PS located in the Bohai Building reached one hundred due to the high resolution of TerraSAR dataset, and these PS were distributed in the whole building from the vertical view. However, there were only eight leveling points, and all were located on the edge of the Bohai Building.

Reasonable and comprehensive comparisons between the different techniques must be assessed using the same spatial locations. Thus, an interpolation of the PS displacement deformation is needed to ensure that InSAR and leveling measurements can be derived for the same locations. 

Considering that the displacement phenomenon has the attribute of spatial persistence, the Inverse Distance Weighted (IDW) interpolation method was used to create a mapping of InSAR displacement for locations corresponding to the leveling points, from the high-density PS sample set. IDW was proposed on the basis of the first principle of geography, which states that if two objects are closer, the features have greater similarity [30]. In the case of the deformation dataset from IPTA analysis, the IDW interpolator was deployed on the basis of discrete PS; the distances between the PS locations and the leveling location were used as weights, and the InSAR displacement was used to estimate the displacement in the corresponding level points [4]. Setting the weight by distance in the PS sample set prevents increasing errors caused by a single point during IDW interpolation, such as facade PS scattered by the roads.

**Figure 9:**
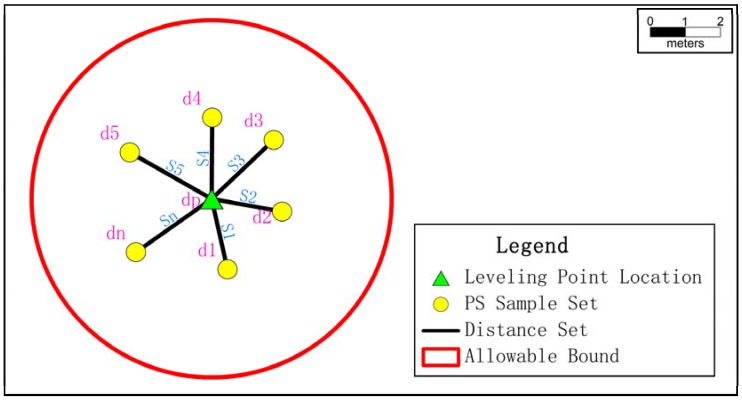
Inverse Distance Weighted (IDW) interpolation to obtain InSAR displacement in the location of leveling points with high-density PS sample set and weights related to distance.

The PS are located in the 3D scene, and the coordinates of each PS can be expressed in term of (x,y,z). During integral analysis of the deformation state, however, a building is usually considered as rigid body without considering elastic deformation, as the selected buildings were made of reinforced concrete, and a rigid motion model can visually describe the deformation state of the building [31,32]. Therefore, the displacement information from the roof best matches displacement information from the bottom of the building with the same values in the *X* direction and *Y* direction, and the spatial difference in the *Z* direction can be eliminated. Thus, in this study, Figure 9 shows the corresponding location of leveling point $P(x_{P},y_{P},d_{P})$ (green triangle), the discrete PS set in a nearby zone $Q_{i}(x_{i},y_{i},d_{i})$ i=1,2,⋯,n (yellow point), and the distance between the discrete PS set and the corresponding leveling point $\left(S_{1},S_{2},\cdots ,S_{n}\right)$ (black line). The weight is related to this distance, and is defined as the reciprocal of distance. Thus, the IDWinterpolation is used to achieve the displacement property $d_{P}$ in P through the following Equation:

(1) $$d_{p}=\frac{\frac{1}{S_{1}}}{\frac{1}{S_{1}}+\frac{1}{S_{2}}+\ldots +\frac{1}{S_{n}}}d_{1}+\frac{\frac{1}{S_{2}}}{\frac{1}{S_{1}}+\frac{1}{S_{2}}+\ldots +\frac{1}{S_{n}}}d_{2}+\ldots +\frac{\frac{1}{S_{n}}}{\frac{1}{S_{1}}+\frac{1}{S_{2}}+\ldots +\frac{1}{S_{n}}}d_{n}$$

### 3.3. Validation Method

To quantitatively demonstrate the millimeter accuracy of InSAR in practical building applications, both Ordinary Least Squares (OLS) regression analysis from a viewpoint of mathematical analysis and statistical measurement error analysis based on the consideration of the survey are proposed, and used for further comparison in following sections. 

#### 3.3.1. OLS Regression Analysis

The OLS method is used in regression analysis to identify a connection between these two interdependent variables from the observation of many points. The most common model is linear regression, which is defined as:

(2) y=α+βx

where x and y are two variables, and α and β are the unknown parameters. In our case, x is the displacement value surveyed in leveling, and y is the displacement value extracted from InSAR.

There are two parameters using to evaluate the quality of the regression analysis, the correlation coefficient and the estimated standard deviation respectively. The correlation coefficient describes the degree of fit between the regression model and the observations, where a value near 1 indicates that the degree of fit is closer. The estimated standard deviation in OLS regression analysis is expressed as Root Mean Square Error (RMSE) of $S_{y}$, a smaller value always corresponds to regression analysis results that more closely fit the model. It is defined as the amount of absolute deviation between the variable y and the regression model $\overset{\wedge }{y}=\overset{\wedge }{\alpha }+\overset{\wedge }{\beta }x$:

(3) $$S_{y}=\sqrt{\frac{\sum {\left(y_{i}-\overset{\wedge }{y_{i}}\right)}^{2}}{n-2}}$$

#### 3.3.2. Statistical Analysis of Measurement Error

Measurement error can be defined as the difference between observation and true value. In this study, the true displacement information for the two buildings is unknown. To validate the InSAR accuracy in buildings, classical leveling displacement with high precision was considered as the true deformation [33,34]. A statistical analysis of the difference between InSAR ($d_{insar}$) and leveling ($d_{level}$) was executed using the RMSE index of δ. In the case of limited observations (n), δ is calculated from following equation:

(4) $$\delta =\pm \sqrt{\frac{\left(d_{insar}-d_{level})(d_{insar}-d_{level}\right)}{n}}$$

## 4. Validation

### 4.1. Case 1: The Bohai Building

Results from the final analysis of displacement in the Bohai Building are presented in Table 4. For every leveling point in the Bohai Building, there are both cumulative displacements measured by the leveling (second row in Table 4) and the displacement extracted from InSAR data (third row in Table 4). They were compared using OLS regression and a statistical analysis of measurement error as follows.

**Figure 10:**
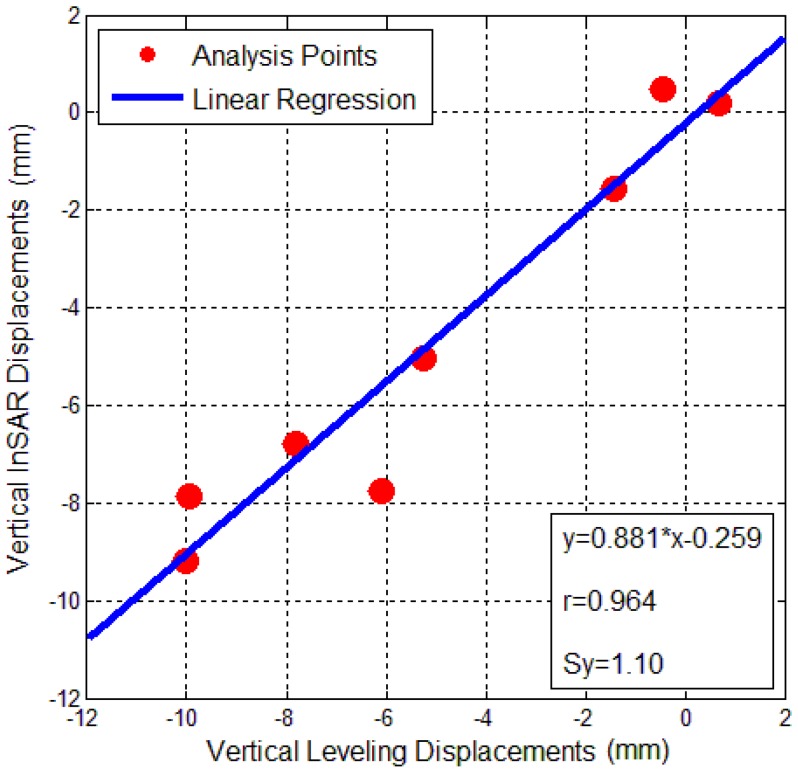
Ordinary Least Squares (OLS) Regression analysis in vertical displacements of the Bohai Building between extracted from InSAR and surveyed from leveling.

OLS regression result between InSAR and leveling displacement of the Bohai Building is shown in Figure 10. For these eight points in the figure, the *x*-axis represents displacement from leveling, and the *y*-axis indicates displacement estimated by InSAR. The blue line represents the linear regression result. The eight red points are near this line, which shows that the regression equation y=0.881x−0.259 fits these data closely. The deviation from the linear relation of was approximately 12% with a bias of approximately 0.259 mm. The linear correlation coefficient value r is 0.964 (very close to the constant of 1), implying that the fit between InSAR measurements and leveling of the Bohai Building is very high. The estimated standard deviation $S_{y}$ was 1.10 mm, indicating that the InSAR accuracy in the Bohai Building reached the accuracy of nearly 1 mm.

**Figure 11:**
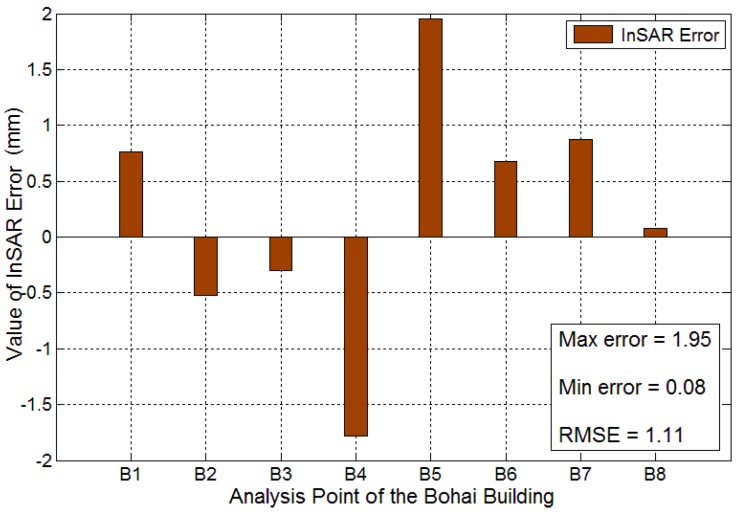
Measurement error between the extracted InSAR displacement and the truth leveling data in the Bohai Building, bigger column correspond to larger error.

Measurement from classical leveling is used as a true deformation value to analyze the InSAR error in the Bohai Building; the measurement error results for the InSAR deformation values are reported in Figure 11. The maximum error is located in B5 with a value of 1.95 mm, and the location of minimum error is B8 with a value of 0.08 mm. An analysis of the eight error values using Equation (4) shows that the RMSE of δ is 1.11 mm, indicating that the accuracy of InSAR for the Bohai Building reached the accuracy of near 1 mm. 

### 4.2. Case 2: The China Theater

The displacement results for the China Theater are presented in Table 5. For each point in the China Theater, the measured displacements from InSAR and leveling were also compared with the OLS regression and statistical error of measurement values.

**Figure 12:**
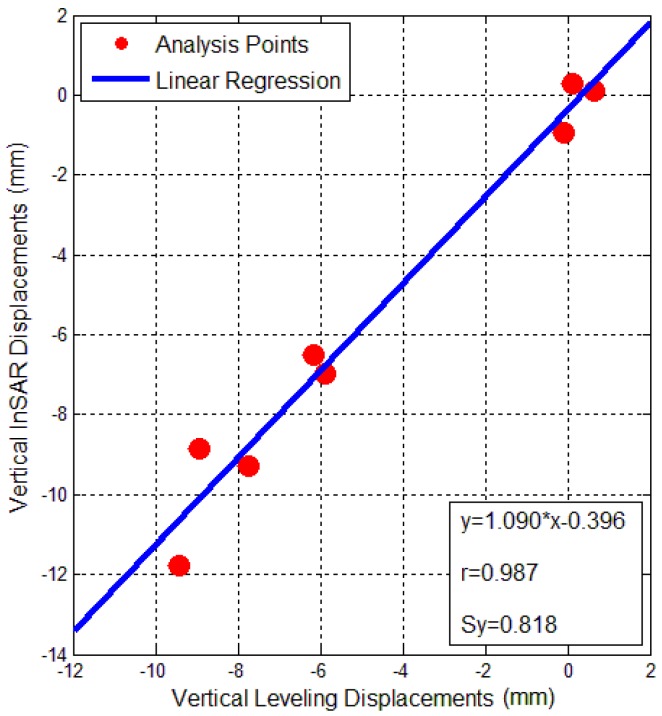
OLS Regression analysis in vertical displacements of the China Theater between extracted from InSAR and surveyed from leveling.

OLS regression result between InSAR and leveling displacement of the China Theater is displayed in Figure 12. The *x*-axis, *y*-axis and blue line have the same meaning as Figure 10, and all eight red points are near the blue regression line, indicating that the regression model y=1.09x−0.369 fits closely to the monitoring results. This linear model indicates a deviation from the linear relation of approximately 9% and a bias of approximately 0.369 mm. The linear correlation coefficient value r from the China Theater was 0.987, and very close to the constant of 1, which implies that the linear regression between InSAR and leveling in the China Theater is very high. The larger value suggests that the OLS regression results from the China Theater were better than those for the Bohai Building. The estimated standard deviation $S_{y}$ from the China Theater was 0.818 mm, a value significantly lower than 1 mm, meaning that the accuracy of InSAR for the China Theater was even be better than 1 mm.

**Figure 13:**
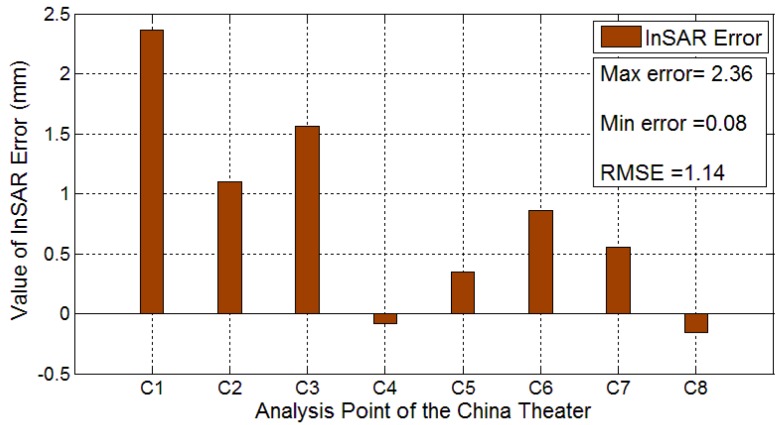
Measurement error between the extracted InSAR displacement and the truth leveling data in the China Theater, bigger column correspond to larger error.

The high accuracy leveling measurements were used as a true value of displacement when analyzing the InSAR error of measurement for the China Theater. The final InSAR error results are reported in Figure 13. The maximum error is located in C1 with a value of 2.36 mm, and the location of minimum error is C4 with a value of 0.08 mm. The RMSE of δ is 1.11 mm, calculated from Equation (4) and the eight error values, which also indicates that the accuracy of InSAR in the China Theater reaches nearly 1 mm.

## 5. Discussion

### 5.1. Analysis of the Validated Results

To evaluate the building displacement results estimated using InSAR technique, we compare them with level of displacement measurements covering approximately the same period as that of the SAR imagery using OLS regression analysis and error of measurement. Because these two validation methods were proposed based on different theory, there are disparity between accuracy conclusion. Table 6 shows results of the two RMSE indexes for the two selected buildings.

In the Bohai Building, OLS regression results are similar to result from the measure error analysis. Nevertheless, for the China Theater, there is a sharp difference between these two measures. The accuracy of the OLS regression results is better than the measure error analysis with a value of 0.32 mm. Table 7 presents a comparison of the cumulative displacements between InSAR and leveling. The first row shows point IDs, the second row gives the in-situ level results, the third row gives InSAR results after updating with the linear model of y=1.09x−0.369; the fourth row gives the displacement differences between the InSAR results and level results after regression analysis; the fifth row gives the InSAR results before updating; and the sixth row gives the direct displacement differences between the InSAR results and the leveling results. 

The differences between regression analysis error and measurement error are analyzed to improve their accuracy. The maximum error of 2.36 mm for measure error was reduced to 1.11 mm after OLS regression. In addition, the error of 1.56 mm in C3was also reduced to 0.47 mm after OLS regression. Therefore, the maximum error after regression analysis became 1.28 mm, and the errors for many points were also reduced. Thus, the InSAR monitoring accuracy was improved.

However, in actual application, because of the difficulties in acquiring in-situ leveling data for each building and the particularity of the update model, the OLS regression analysis method is limited to the following applications: (1) validating the close correlations between InSAR and leveling; (2) improving the monitoring accuracy of InSAR in relevant buildings; and (3) identifying the highest possible precision through the InSAR technique. Meanwhile, the error measure analysis is not limited by external conditions, thus the accuracy result from it can be used in our subsequent analyses and applied to other perennial InSAR applications.

According to Table 6, the RMSE from the statistical analysis of the measurement error in the Bohai Building was 1.11 mm, while the corresponding RMSE for the China Theater is 1.14 mm. These two values are in intervals of (0.7 mm, 2.1 mm); thus, these experiments demonstrate that InSAR can achieve third level accuracy listed in Table 1, and can be used in the primary assessments of building safety. 

### 5.2. Strengths and Weaknesses of the InSAR Technique in Monitoring Buildings

We compared building displacement estimated from InSAR to estimates derived by leveling in the three following aspects. (1) Covering area: One high-resolution SAR covers a region of more than 1000 km^2^, whereas leveling covers only some points in one monitoring task. (2) High density: SAR not only monitors building with more than one hundred points in one building, but also monitors a majority of the building in per unit area. Leveling, however, usually monitors ten points in one building, and only some buildings are chosen for monitoring and analysis per unit area. (3) Monitoring accuracy: Traditionally, leveling accuracy for buildings is at the second level, whereas InSAR accuracy is higher than the third level and lower than the second level.

Therefore, considering these three aspects, the best way to monitor buildings is to integrate InSAR with leveling. One possible solution is that, after using InSAR to obtain a census of building safety, buildings determined to be dangerous will be monitored with precise leveling.

## 6. Conclusions

A valuation experiment in Tianjin City’s center was conducted to determine if building deformation monitoring from InSAR can indeed reach the same millimeter level accuracy as the leveling technique. Ten TSX images, spanning over six months, were used through in IPTA analysis to generate a displacement map of annual velocity, and deformation information for two landmark buildings, the Bohai Building and the China Theater, was extracted. The true displacement values of these two buildings were acquired concurrently by setting leveling points and high accuracy monitoring. 

After a direction diversity process with proper assumption and a spatial diversity process by IDW interpolation, the InSAR results were compared to the leveling using OLS regression and measure error analyses. In the Bohai Building, a linear regression analysis revealed a very high correlation (0.964) between InSAR processing results and the ground leveling record, with an RMSE index ($S_{y}$) of 1.10 mm. Moreover, lower RMSE index of δ was acquired with a value of 1.11 mm. Results obtained by applying these evaluation indexes to the China Theater were 0.987 (correlation index), 0.818 mm ($S_{y}$), and 1.14 mm (δ), respectively. Our validation test found that InSAR technique adapted for buildings can reach millimeter-level accuracy.

As a further InSAR application for monitoring buildings, we discussed the difference between OLS regression and measurement error analyses with the example of the China Theater, and the usability of these two methods in practical application. Then, a conclusion that InSAR can achieve third level accuracy and be used in primary assessments of building safety is achieved. Thus, with the advantages of covering large area and high densities, the comprehensive utilization of InSAR and leveling is proposed for further application of monitoring building deformation.

## Figures and Tables

**Table 1:** Monitoring levels and accuracy of building.

Deformation Monitoring Levels	Monitoring Accuracy	Applicability
First	0.2 mm	Monitoring important ancient buildings
Second	0.7 mm	Monitoring buildings with foundation design of A and B
Third	2.1 mm	Monitoring buildings with foundation design of B and C

**Table 2:** Main features of the available SAR dataset in the AOI of Tianjin.

Feature	TerraSAR	Feature	TerraSAR
Wavelength	X (~3.1 cm)	Resolution	3 m
Incidence Angle	37.3°	Number of images	10
Geometry	Ascending	Cover Area	30 km × 50 km
Image Model	Strip Map		

**Table 3:** Displacement results of two building from leveling (mm).

Monitoring Points	Displacements	Monitoring Points	Displacements
B1	−0.29	C1	−11.77
B2	0.7	C2	−6.97
B3	−1.28	C3	−9.30
B4	−5.96	C4	−8.84
B5	−9.81	C5	−6.51
B6	−9.88	C6	−0.94
B7	−7.67	C7	0.10
B8	−5.12	C8	0.30

**Table 4:** The final displacements from leveling and InSAR in Bohai Building (mm).

Point	Leveling Results	InSAR Results
B1	−0.29	0.47
B2	0.7	0.18
B3	−1.28	−1.58
B4	−5.96	−7.74
B5	−9.81	−7.86
B6	−9.88	−9.2
B7	−7.67	−6.8
B8	−5.12	−5.04

**Table 5:** The final displacements from leveling and InSAR in China Theater (mm).

Point	Level Results	InSAR Results	Point	Level Results	InSAR Results
C1	−11.77	−9.42	C5	−6.51	−6.16
C2	−6.97	−5.88	C6	−0.94	−0.08
C3	−9.30	−7.74	C7	0.10	0.65
C4	−8.84	−8.92	C8	0.30	0.14

**Table 6:** Different Root Mean Square Error (RMSE) analysis results.

Building	RMSE Index (Regression Analysis)	RMSE Index (Measure Error)
Bohai Building	1.10 mm	1.11 mm
China Theater	0.82 mm	1.14 mm

**Table 7:** Detailed analysis between regression analysis and measure error (mm).

Point	Level Result	InSAR Result (Regression Analysis)	Regression Analysis Error	InSAR Result (Measure Error)	Measure Error
C1	−11.77	−10.66	1.11	−9.42	2.36
C2	−6.97	−6.80	0.17	−5.88	1.10
C3	−9.30	−8.83	0.47	−7.74	1.56
C4	−8.84	−10.12	−1.28	−8.92	−0.08
C5	−6.51	−7.11	−0.60	−6.16	0.35
C6	−0.94	−0.48	0.46	−0.08	0.86
C7	0.10	0.31	0.21	0.65	0.55
C8	0.30	−0.24	−0.54	0.14	−0.16

## References

[1] Ciampalini A., Bardi F., Bianchini S., Frodella W., del Ventisette C., Moretti S., Casagli N. (2014). Analysis of building deformation in landslide area using multisensor PSInSAR™ technique. Int. J. Appl. Earth Obs. Geoinform..

[2] Tofani V., Raspini F., Catani F., Casagli N. (2013). Persistent scatterer interferometry (PSI) technique for landslide characterization and monitoring. Remote Sens..

[3] Frattini P., Crosta G.B., Allievi J. (2013). Damage to buildings in large slope rock instabilities monitored with the PSInSAR™ technique. Remote Sens..

[4] Bianchini S., Pratesi F., Nolesini T., Casagli N. (2015). Building deformation assessment by means of persistent scatterer interferometry analysis on a landslide-affected area: The Volterra (Italy) case study. Remote Sens..

[5] Pratesi F., Tapete D., Terenzi G., Del V.C., Moretti S. Structural Assessment of Case Study Historical and Modern Buildings in the Florentine Area Based on a PSI-Driven Seismic and Hydrogeological Risk Analysis. Engineering Geology for Society and Territory, Proceedings of the 2014 IAEG, Turin, Italy, 15–19 September 2014.

[6] Qing G.D., Ling Z., Yan W., Man L., Bin L. (2014). Monitoring subsidence on Shanghai Metro line ten during construction and operation using high-resolution InSAR. Shanghai Land Resour..

[7] Wang D., Lu X.Z., Zhang Z.J., Pan Q.L., Wang S.L., Wang B.F., Liu G.Y., Zhang F.L., Yan X.P., Ou H.P. (2007). JGJ8–2007, Code for Deformation Measurement of Building and Structure.

[8] Zebker H.A., Villasenor J. (1992). Decorrelation in interferometric radar echoes. IEEE Trans. Geosci. Remote Sens..

[9] Ferretti A., Prati C., Rocca F. Permanent Scatterers in SAR Interferometry. Proceeding of the 1999 IEEE International Geoscience and Remote Sensing Symposium (IGARSS).

[10] Ferretti A., Prati C., Rocca F. (2001). Permanent scatterers in SAR interferometry. IEEE Trans. Geosci. Remote Sens..

[11] Beradino P., Fornaro G., Lanari R., Sansosti E. (2002). A new algorithm for surface deformation monitoring based on small baseline differential SAR interferograms. IEEE Trans. Geosci. Remote Sens..

[12] Mora O., Mallorqui J.J., Broquetas A. (2003). Linear and nonlinear terrain deformation maps from a reduced set of interferometric SAR images. IEEE Trans. Geosci. Remote Sens..

[13] Werner C., Wegmüller U., Strozzi T., Wiesmann A. Interferometric Point Target Analysis for Deformation Mapping. Proceedings of the IEEE International Geoscience and Remote Sensing Symposium.

[14] Kumar V., Venkataramana G., Høgda K. (2011). Glacier surface velocity estimation using SAR interferometry technique applying ascending and descending passes in Himalayas. Int. J. Appl. Earth Obs. Geoinform..

[15] Lanari R., Lundgren P., Sansosti E. (1998). Dynamic deformation of Etna volcano observed by satellite radar interferometry. Geophys. Res. Lett..

[16] Lubitz C., Motagh M., Wetzel H., Kaufmann H. (2013). Remarkable urban uplift in Staufen IM Breisgau, Germany: Observations from TerraSAR-X InSAR and leveling from 2008 to 2011. Remote Sens..

[17] Ge L., Chang H.C., Rizos C. (2007). Mine subsidence monitoring using multi-source satellite SAR images. Photogramm. Eng. Remote Sens..

[18] Wegmuller U., Walter D., Spreckels V., Werner C. (2010). Nonuniform ground motion monitoring with terraSAR-X persistent scatterer interferometry. IEEE Trans. Geosci. Remote Sens..

[19] Luo Q.L., Perissin D., Zhang Y.Z., Jia Y.L. (2014). Perissin, L- and X-Band multi-temporal InSAR analysis of Tianjin subsidence. Remote Sens..

[20] Qin Y.X., Perissin D. (2015). Monitoring ground subsidence in Hong Kong via spaceborne radar: Experiments and validation. Remote Sens..

[21] Bru G., Herrera G., Tomás R., Duro J., Dela V.R., Mulas J. (2010). Control of deformation of buildings affected by subsidence using persistent scatterer interferometry. Struct. Infrastruct. Eng..

[22] Arangio S., Calò F., Di Mauro M., Bonano M., Marsella M., Manunta M. (2014). An application of the SBAS-DInSAR technique for the Assessment of structural damage in the city of Rome. Struct. Infrastruct. Eng..

[23] Ketelaar G. (2009). Satellite Radar Interferometry Subsidence Monitoring Techniques. Remote Sensing and Digital Image Processing.

[24] Wegmuller U., Soreckel V., Werner C., Strozzi T., Wiesmann A. Monitoring of mining induced surface deformation using L-band SAR interferometry. Proceedings of the 2005 IEEE International Geoscience and Remote Sensing Symposium, IGARSS ’05.

[25] Wegmuller U., Werner C., Strozzi T., Wiesmann A. Monitoring Mining Induced Surface Deformations. Proceedings of the 2004 IEEE International Geoscience and Remote Sensing Symposium, IGARSS ’04.

[26] Hooper A. (2008). A multi-temporal InSAR method incorporating both persistent scatterer and small baseline approaches. Geophys. Res. Lett..

[27] Lan H.X., Li L.P., Liu H.J., Yang Z.H. (2012). Complex urban infrastructure deformation monitoring using high resolution TerraSAR-X PSI. IEEE J. Sel. Top. Appl. Earth Obs. Remote Sens..

[28] Hanssen R.F. (2002). Radar Interferometry-Data Interpretation and Error Analysis.

[29] Perissin D., Ferretti A. (2007). Urban-target recognition by means of repeated spaceborne SAR images. IEEE Trans. Geosci. Remote Sens..

[30] Tobler W. (1970). A computer movie simulating urban growth in the Detroit region. Econ. Geogr..

[31] Eurocode—Basis of Structural Design. http://www.unirc.it/documentazione/materiale_didattico/599_2010_260_7481.pdf.

[32] Ricceri G., Soranzo M. (1985). An analysis on allowable settlement of structures. Riv. Ital. Geotec..

[33] Chen J., Wu J. (2013). Deformation Trend Extraction Based on Multi-Temporal InSAR in Shanghai. Remote Sens..

[34] Zhang Y.H., Wu H.A., Kang Y.H., Zhu C.G. (2016). Ground Subsidence in the Beijing-Tianjin-Hebei Region from 1992 to 2014 Revealed by Multiple SAR Stacks. Remote Sens..

